# Engineering 3D Printed Microfluidic Chips for the Fabrication of Nanomedicines

**DOI:** 10.3390/pharmaceutics13122134

**Published:** 2021-12-10

**Authors:** Aytug Kara, Athina Vassiliadou, Baris Ongoren, William Keeble, Richard Hing, Aikaterini Lalatsa, Dolores R. Serrano

**Affiliations:** 1 Pharmaceutics and Food Technology Department, School of Pharmacy, Universidad Complutense de Madrid, Plaza Ramón y Cajal s/n, 28040 Madrid, Spain; akara@ucm.es (A.K.); bongoren@ucm.es (B.O.); 2 Biomaterials, Bio-engineering and Nanomedicine (BioN) Lab, Institute of Biomedical and Biomolecular Sciences, School of Pharmacy and Biomedical Sciences, University of Portsmouth, White Swan Road, Portsmouth PO1 2DT, UK; Athina.Vassiliadou@myport.ac.uk; 3 School of Mechanical and Design Engineering, University of Portsmouth, Portsmouth PO1 3DJ, UK; william.keeble@port.ac.uk; 4 Electron Microscopy and Microanalysis Unit, School of the Environment, Geography and Geoscience, University of Portsmouth, Portsmouth PO1 3QL, UK; richard.hing@port.ac.uk; 5 Facultad de Farmacia, Instituto Universitario de Farmacia Industrial, Universidad Complutense de Madrid, 28040 Madrid, Spain

**Keywords:** microfluidics, 3D printing, nanomedicines, nifedipine, SLA, FDM, stereolithography

## Abstract

Currently, there is an unmet need to manufacture nanomedicines in a continuous and controlled manner. Three-dimensional (3D) printed microfluidic chips are an alternative to conventional PDMS chips as they can be easily designed and manufactured to allow for customized designs that are able to reproducibly manufacture nanomedicines at an affordable cost. The manufacturing of microfluidic chips using existing 3D printing technologies remains very challenging because of the intricate geometry of the channels. Here, we demonstrate the manufacture and characterization of nifedipine (NFD) polymeric nanoparticles based on Eudragit L-100 using 3D printed microfluidic chips with 1 mm diameter channels produced with two 3D printing techniques that are widely available, stereolithography (SLA) and fuse deposition modeling (FDM). Fabricated polymeric nanoparticles showed good encapsulation efficiencies and particle sizes in the range of 50–100 nm. SLA chips possessed better channel resolution and smoother channel surfaces, leading to smaller particle sizes similar to those obtained by conventional manufacturing methods based on solvent evaporation, while SLA manufactured nanoparticles showed a minimal burst effect in acid media compared to nanoparticles fabricated with FDM chips. Three-dimensional printed microfluidic chips are a novel and easily amenable cost-effective strategy to allow for customization of the design process for continuous manufacture of nanomedicines under controlled conditions, enabling easy scale-up and reducing nanomedicine development times, while maintaining high-quality standards.

## 1. Introduction

Nanomedicine is the application of nanotechnology in the medical field with important advances in terms of drug delivery, in vitro and in vivo diagnostics and imaging, regenerative medicine, and local implanted devices. Nanomedicines can be developed either as drug delivery systems or biologically active drug products consisting of at least two components, one of which is the active ingredient [1]. Nanomedicines have spurred significant growth in the drug delivery sector due to their ability to enhance the targeting of therapeutics to the desired site of action, which allows for a reduction in the dose, and consequently, limits the appearance of side-effects. They have found significant applications, particularly in the treatment of cancer, pain and infectious diseases. The global nanomedicine market is anticipated to reach USD 350.8 billion by 2025 [2], while licensing of nanomedicines is rising as demonstrated by the fact that in 2016, the FDA and EMA had approved more than 25 nanomedicines, while more than 45 were still under study. New applications in vaccinations as demonstrated by the formulation of mRNA vaccines in the recent COVID-19 pandemic is also currently contributing to the growth of the market [3]. Thus, nanomedicines have raised significant interest and investment from researchers and investors worldwide during the last decade [4,5,6,7,8,9].

The European Medicine Agency’s (EMA) current regulatory framework focuses on the benefit/risk ratio in their licensing [10,11,12], which requires that nanomedicines are subjected to toxicology and ecotoxicology studies as well as remain under pharmacovigilance once marketed. The FDA has no specific regulatory framework for nanomedicines, but has recently drafted guidance for industry and special guidance for liposomal nanomedicines that are leading the entry into the market [4]. Although the FDA does not define nanoparticles or clearly separate biological products in the nanometer scale, when considering whether a product involves the application of the nanotechnology, it assesses whether a material or end product is engineered to have a least one external dimension, or an internal or surface structure in the nanoscale range (1–100 nm) or whether this material or end product is engineered to exhibit properties or phenomena (physical, chemical, biological) that are attributable to its dimension(s), even if one of these dimensions falls outside the nanoscale range and are up to one micrometer (up to 1000 nm) [5].

The major bottleneck in the uptake of nano-enabling technologies in the market is due to three main challenges: (1) technical issues, including reliance on batch manufacturing; (2) lack of a clear legislative framework; and (3) economic risks as R&D is carried out mainly by small and medium-sized enterprises as big industries do not want to take risk on projects that have not yet been validated [13]. Once their potential and feasibility are demonstrated, Big Pharma is likely to buy SMEs or license the products. Thus, to facilitate their technology readiness and scale-up to first in man studies, successful fabrication of nanomedicines with processes that can be continuous and able to match high-quality standards are critical.

The current preparation method for the majority of licensed nanomedicines remains discontinuous and faces a number of problems such as high batch-to-batch variability, it includes laborious multi-step processes, the need for an expert workforce and is not easily amenable to industrial scale-up involving a complex process control [14,15,16]. Several techniques have emerged in recent years for nanomedicine manufacture; however, a paradigm shift occurred when microfluidic strategies were employed [17]. Microfluidic manufacture of nanomedicines allows for continuous and industrial amenable production, allowing for greater control of the process and facile scale-up. However, the engineering of microfluidic devices remains complex and available to limited manufacturers for microfluidic devices. Traditional fabrication of microfluidic devices involving injection molding using polydimethylsiloxane (PDMS) has failed to address complex control systems (such as nonstandard user interfaces), while costs remain high [18]. Three-dimensional printing technologies have overcome these barriers in other applications and remain a more cost-effective strategy able to provide excellent channel resolution and utilize a range of materials with ideal properties (transparency, non-fluorescent, and biocompatible) [9,19,20,21,22,23]. However, until now, there are very few reports available that demonstrate the manufacturing of 3D printed microfluidic chips for the fabrication of nanomedicines due to their intricate geometry [24].

The American Society for Testing and Materials (ASTM) Committee F42 on Additive Manufacturing Technologies divided 3D printers into seven categories according to their working principles [25]: vat photopolymerization, powder bed fusion, binder jetting, material jetting, material extrusion, sheet lamination, and directed energy deposition. The type of 3D printing method that is chosen for a particular application type has significant implications and depends on the chosen material type, material compatibility, material availability, size, resolution, yield, speed, and how the final object slicing process takes place [26,27,28]. We have selected the most widely available techniques such as fused deposition modeling (FDM), a type of material extrusion, and stereolithography (SLA), a type of vat polymerization, as additive manufacturing technologies to fabricate microfluidic chips with adequate resolution to enable nanomedicine fabrication. Due to its high performance at a low cost and the increasingly wide availability of FDM printers, FDM is a reasonable first option, while SLA print qualities are of high resolution and closely resemble the available microfluidic manufacturing techniques. Pharmaceutical grade Eudragit L-100 was selected as a biocompatible polymer for oral controlled drug delivery in our study, while nifedipine, a BCS Class II drug, was selected as a model drug [29]. Here, we demonstrate the encapsulation of nifedipine within polymeric nanoparticles using our customized design and FDM and SLA 3D printed microfluidic chips, and compare the key quality manufacturing attributes to those of conventional manufacturing processes for nanoparticles, such as solvent evaporation. Drug encapsulation, particle size, and zeta potential of the fabricated nanoparticles were evaluated and compared to standard manufacturing methods for nanoparticle production, such as solvent evaporation and release studies were also undertaken to assess the in vitro performance of manufactured oral nanomedicines. Thus, we demonstrated that microfluidic manufacture enabled easy fabrication of chips within laboratory settings in academia or SMEs to pave the way for easy and cost-effective manufacture and scale-up of continuous processes for manufacturing nanomedicines.

## 2. Materials and Methods

### 2.1. Materials

Nifedipine (>98%, NFD) was obtained from Kemprotec Limited (Carnforth, UK), while Eudragit L-100-55 (methacrylic acid-ethyl acrylate copolymer 1:1) was donated by Evonik Rohm GmbH (Essen, Germany). Cyclic olefin copolymer filaments (transparent, 1.75 mm diameter size) (COC) were obtained from Creamelt^®^ 3D Printing Materials (Rapperswil-Jona, Switzerland) and UV polymerizable resin (405 nm) was obtained from Anycubic^®^ (Shenzhen, China). Deoxycholic acid sodium salt (>98%) was obtained from Fluka Biochemica (Cleveland, OH, USA). Solvents such as methanol, ethanol, dimethylsulfoxide, and other chemicals such as sodium chloride, potassium chloride, disodium hydrogen phosphate, potassium dihydrogen phosphate, and polysorbate 80 (Tween 80) were all of ACS grade or above and were purchased from Panreac (Barcelona, Spain). Cellulose acetate dialysis membranes with a molecular weight cut-off of 3500 Da were purchased from Medicell Ltd. (London, UK). The Anycubic Photon Mono X (LCD-based SLA printer, 405 nm light source, 0.05 mm 3840 × 2400 XY resolution, 0.01 mm Z resolution, 192 × 120 × 245 mm build volume) and Anycubic Mega Zero (FDM printer, 0.1 mm layer resolution 0.125 mm XY resolution, 0.4 mm nozzle diameter) were obtained from Anycubic^®^ (Shenzhen, China).

### 2.2. Microfluidic Chip Manufacturing and Characterization

The microfluidic chips were designed using Tinkercad^®^ (Autodesk^®^, Mill Valley, CA, USA) with a total length of 8.2 cm, a depth of 3.5 cm, and a height of 0.7 cm. The microfluid chip consisted of two different channels of 2 cm length and 1 mm diameter up to the junction of the inlets. The total length of the channel was 44 cm with 1 mm diameter. The 2-inlet and 1-outlet microfluidic chip designed using Tinkercad^®^ was exported into a standard tessellation language (.stl) digital file. This file was imported into Anycubic Photon Slicer Software (Anycubic^®^, Shenzhen, China) or Ultimaker Cura (Ultimaker B.V.^®^, Utrecht, The Netherlands). The stl. file was sliced to g-code file format for FDM printing and pwmx for SLA printing.

The Anycubic Mega Zero FDM printer melted the COC filament at 245 °C. This filament was extruded with the help of a 0.4 mm diameter nozzle and adhered to the printing platform upon extrusion. While slicing, a file raft was used to avoid damage to the microfluidic chips during printing. Layer height was set to 0.1 mm for achieving high resolution and the printer head followed a geometric pattern with a 10 mm/s speed while printing and 30 mm/s while traveling. The infill was set at 100% and a total number of 70 layers were printed.

The Anycubic^®^ Photon Mono X SLA printer was used to print microfluidic chips under photopolymerization of the Anycubic^®^ UV sensitive resin (transparent yellow) at 405 nm. The solidified resin adhered initially to the metal platform and the other layers adhered to this first layer, thus creating the desired object. Each layer was 0.05 mm thick. The first eight layers were exposed to UV light for a longer time than the others (60 s) to ensure good attachment to the metallic platform. The remaining layers were exposed to UV light for 3 s. At the end of each layer, the UV light was turned off for 1 s, preventing unwanted parts from solidifying. A total number of layers required for the formation of the microfluidic chip using the SLA printer was 140.

#### 2.2.1. Imaging

After printing, the geometry of the chips was visualized with a ZEISS Primo Star microscope (Carl Zeiss Microscopy, White Plains, NY, USA) eyepiece magnification 10×, objective magnification 4×. Chips were also attached with double adhesive tapes and adhered on SEM stubs and imaged under variable pressure using a Zeiss Evo MA10 scanning electron microscope (Carl Zeiss Ltd., Cambridge, UK).

#### 2.2.2. Channel Surface Roughness

Microfluidic chips were printed at 50% of the total height leaving the channel surface exposed. The surface roughness of the channels was measured using a Formtracer SV-C3200 instrument and analyzed using the FORMTRACEPAK software (Mitutoyo UK Ltd., Andover, UK). The measured length was 15 mm at a speed of 0.5 mm s^−1^. The z axis range was set at 800 μm. The arithmetic average roughness (*Rav*) was calculated using the following Equation (1):

(1) $$Rav=\frac{\sum_{n=1}^{N}IZn-ZI}{N}$$

where *Zn* is the individual height value of the measurement point by the laser reflection measurement, *Z* is the mean value of all of the height data points, and *N* is the number of measurement points [30]. The absolute vertical distance between the maximum peak height and the maximum valley depth along the sampling length was expressed as Rz. For statistical comparisons, the Student t-test (Microsoft Excel 2017) was used.

#### 2.2.3. Manufacturing of Nifedipine Polymeric Nanoparticles with 3D Printed Microfluidic Chips

Nifedipine polymeric nanoparticles were prepared by mixing the organic phase, comprised of Eudragit L100-55 (30 mg) and NFD (10 mg) dissolved in ethanol (2 mL) with the aqueous phase, comprised of Tween 80 in deionized water (0.25% *w*/*v*, 20 mg in 8 mL). Both phases were loaded into 10 mL syringes connected to two syringe pumps (New Era Pump Systems, Farmingdale, NY, USA). Each syringe was fitted with a 14-gauge olive-colored needle (with an outer diameter of 1.83 mm) (Fisher Scientific, Madrid, Spain). One end of the silicone tubes was connected to the syringe needle outlet, while the other end was connected to a 20-gauge pink-colored needle (0.91 mm outer diameter), which was attached to the microfluidic chip.

The flow rate of the organic phase was set at 0.5 mL min^−1^ and the flow rate of the aqueous phase was 2 mL min^−1^ to elicit the 1:4 *v/v* ratio when mixed in line. Prior to manufacture of any nanoparticles, the chips were eluted for 30 min to remove any impurities. Similar flow rates and organic to aqueous ratio were used to produce nanoparticles and no NFD was used when blank nanoparticles were prepared. The eluate (10 mL) was collected and rota-evaporated for 10 min (Rotavapor^®^ R-300 (BUCHI Labortechnik AG, Flawil, Switzerland) to remove the ethanol under vacuum (100 mm Hg) at 150 rpm and 60 °C. The formulation was then centrifuged at 5000 rpm for 5 min (Neuation Technologies, Gujarat, India) to remove any free NFD and the supernatant was collected for further analysis. Three milliliters of the supernatant were lyophilized (Lyoquest, Telstar^®^, Madrid, Spain) over 24 h at 0.2 mbar pressure to obtain a solid powder formulation. Both the liquid and solid formulations were stored at 2–8 °C until further analysis under desiccated conditions.

### 2.3. Preparation of Nifedipine Polymeric Nanoparticles with Solvent Evaporation

Eudragit L100-55 (30 mg) and NFD (10 mg) dissolved in ethanol (2 mL) were added dropwise in the aqueous phase comprised of Tween 80 in deionized water (0.25% *w*/*v*, 20 mg in 8 mL) under stirring at 500 rpm over 5 min at room temperature. The mixture was rota-evaporated for approximately 5 min at a 60 °C to remove the ethanol and then centrifuged at 5000 rpm for 5 min prior to collecting the supernatant to remove any unencapsulated NFD. Similarly, three milliliters of the supernatant were lyophilized to obtain a solid powder formulation and kept under refrigerated conditions for further analysis, as for microfluidically prepared particles.

### 2.4. Drug Loading

Final formulations (10 μL) were dispersed in 1 mL of deionized water and from these aqueous stocks, a further dilution was performed in ethanol (1 in 5000). The absorbance of the diluted samples was determined at 240 nm using a V-730 UV-Visible Spectrophotometer (JASCO Inc., Easton, PA, USA). A linear calibration curve was constructed between 0.56 and 50 g mL^−1^ of NFD dissolved in ethanol:water (9:1 *v/v*). The absorbance of similarly diluted blank formulations without NFD was removed.

### 2.5. Particle Size and Zeta Potential

Before particle size and zeta potential measurements were performed, formulations (10 μL) were diluted in 1 mL of deionized water (pH 6.5), and after that the samples were measured with a Microtract Zetatrac apparatus (Microtrac, Montgomeryville, PA, USA). All measurements were performed in triplicate (*n* = 3), and the mean and standard deviation were reported. Before sample analysis, a polystyrene PS02001 (10.06% solid) certified standard with a diameter of 25 nm and a particle size distribution within the 15–35 nm range was measured. The polydispersity index (PDI) was calculated as the square of the standard deviation divided by the mean particle diameter. The span value was calculated based on the D_90_, D_50_, and D_10_ values as:

Span = (D_90_ − D_10_)/D_50_ (2)

### 2.6. Transmission Electron Microscopy (TEM)

A drop of the sample was placed onto a Formvar/carbon-coated grid, and the excess sample was blotted off with Whatman No. 1 filter paper. Samples were negatively stained with 1% *w*/*v* uranyl acetate aqueous solution as previously described [31]. TEM was performed using a JEOL 1400 transmission electron microscope (JEOL Ltd., Tokyo, Japan) operating at 120 KV. An AMT digital camera was used to capture the images.

### 2.7. Fourier-Transform Infrared (FTIR) Spectroscopy

FTIR analysis of lyophilized NFD nanomedicines was carried out with a Nicolet Nexus 670–870 (Thermofisher, Madrid, Spain). A wavelength range between 400–4000 cm^−1^ was used. Each sample (1–3 mg) was mixed manually in an agate mortar and pestle with KBr (200 mg). The powder mixture was then compressed into discs using a PerkinElmer hydraulic press set at a pressure of 10 tons for 10 min. Spectragryph (version 1.2.9, Oberstdorf, Germany) software was used for the interpretation of the spectra. Data normalization was performed.

### 2.8. X-ray Powder Diffraction (pXRD)

Solid-state characterization was performed on lyophilized NFD nanoparticles. Powder X-ray analysis was carried out using a Philips^®^X’Pert-MPD X-ray diffractometer (Malvern Panalytical^®^; Almelo, The Netherlands) equipped with Ni-filtered Cu K radiation (1.54). A 40 kV voltage and 40 mA current were used to perform the study. PXRD patterns were recorded at a step scan rate of 0.05° per second from 5° to 40° on the 2-theta scale (*n* = 3) [32].

### 2.9. Differential Scanning Calorimetry (DSC)

DSC scans were performed using nitrogen as the purge gas on a QA-200 TA instrument (TA instruments, Elstree, UK) calorimeter. Lyophilized NFD nanoparticles prepared microfluidically were weighed (4–6 mg) before being sealed in an open aluminum pan. Temperatures were set to range from 10 to 200 °C. The instrument was calibrated using indium as the standard. The glass transition temperatures reported are the transition’s midpoint (*n* = 3).

### 2.10. Release Studies

Release tests were conducted in triplicate using a USP Dissolution Apparatus 2 (Erweka DT 80, Heusenstamm, Germany) at a speed of 50 rpm [29]. Freshly prepared NFD nanoparticles (10 mL, 5 μg mL^−1^) were introduced in a dialysis bag with a molecular weight cut-off of 3500 Da (Medicell, London, UK). USP simulated gastric fluid (SGF) without enzymes (pH 1.2) and USP simulated intestinal fluid (SIF) without enzymes (pH 6.8) were used as dissolution media [19]. The bag was added to SGF (250 mL) for the first two hours after which the bag was transferred to another vessel containing SIF (250 mL) and the pH was adjusted to 6.8 ± 0.05. Media were maintained at 37 ± 0.5 °C and at 5, 10, 15, 30, and 45 min, 1, 2, 3, 4 and 6 h, samples (2 mL) were removed from the dissolution media and filtered through a hydrophilic 0.45 m filter (Millipore, Millex-LCR, MA, USA). NFD was quantified by HPLC using a Thermo BDS Hypersil C18 reverse-phase column (250 × 4.6 mm, 5 µm). The HPLC was comprised of Varian Prostar 230 Solvent Delivery Module, a Varian Prostar autosampler 410, and a Varian Prostar 310 UV-visible detector (Varian, CA, USA). Integration of the peaks was performed with a Galaxie Chromatography Data System (Varian, CA, USA). The mobile phase consisting of methanol:water:acetonitrile (36:55:9, *v/v*) was pumped at a flow rate of 1 mL min^−1^, and the sample injection volume was set at 40 µL. The column temperature was kept at 25 °C and the detector was set at 240 nm. A linear calibration curve was obtained between 50–0.1 µg mL^−1^ (y = 0.0598x − 0.038, R^2^ = 0.9999). The method used was previously validated with a detection limit of 0.12 μg/mL, while the quantification limit was 0.4 μg mL^−1^ [33].

The dissolution data obtained were fitted [34,35] using the zero order (Equation (3)), first order (Equation (4)), Hixson–Crowell (Equation (5)), Korsmeyer–Peppas (Equation (6)) and Higuchi (Equation (7)) kinetic equations:

*Q_t_* = *Q*_0_ + *K*_0_*t* (3)

*LogQ_t_* = *logQ*_0_ + *K*_1_*t*2.303 (4)

*W*_0_^1/3^ − *W*^1/3^_*t*_ = *K_s_t* (5)

*Log(Mt/M∞)* = *log K_kp_* + *nlogt* (6)

*Q* = *tDC_s_*(2*C* − *C_s_*) (7)

where *Q_t_* is the amount of drug dissolved in time *t*, *Q*_0_ is the initial amount of drug in the solution (most times, *Q*_0_ = 0), *W*_0_ is the initial amount of drug in the tablet, *W_t_* is the remaining amount of drug in the tablet; *Mt*/*M*∞ is the fraction of drug release at time *t*; *D* is the diffusion constant, *C* is the initial drug concentration, *C_s_* is the drug solubility in the matrix medium and *Q* is the amount of drug release per time, *t*, per unit area; *K*_1_ is the first order release constant, *K*_0_ is the zero order release constant, *K_s_* is a constant incorporating the surface-volume relation; *K_kp_* is a constant that describes the structural and geometric characteristics of the drug dosage form; *n* is the release exponent which describes the drug release mechanism. The *n* has a value of 0.5, 0.45 or 0.43 when the particle shape is a thin film, a cylinder or a sphere, respectively, which indicates Fickian release controlled by diffusion [32]. Anomalous non-Fickian transport is observed when *n* is between those values and 1 (0.5 < *n* < 1 for thin films, 0.45 < *n* < 1 for cylinder and 0.43 < *n* < 1 for spheres). Values of *n* = 1 correspond to zero order release [36]. The choice of release profile that best fits the release data is determined based on the obtained regression coefficient (R^2^) [32,34,37]. Repeated measures ANOVA with a Tukey’s post-hoc test was utilized to compare release profiles (GraphPad Prism 9, San Diego, CA, USA) over time for the FDM and SLA microfluidically prepared and conventionally prepared with solvent evaporation nifedipine-loaded nanoparticles.

## 3. Results

### 3.1. Design and Engineering of 3D Printed Microfluidic Chips

**Figure 1:**
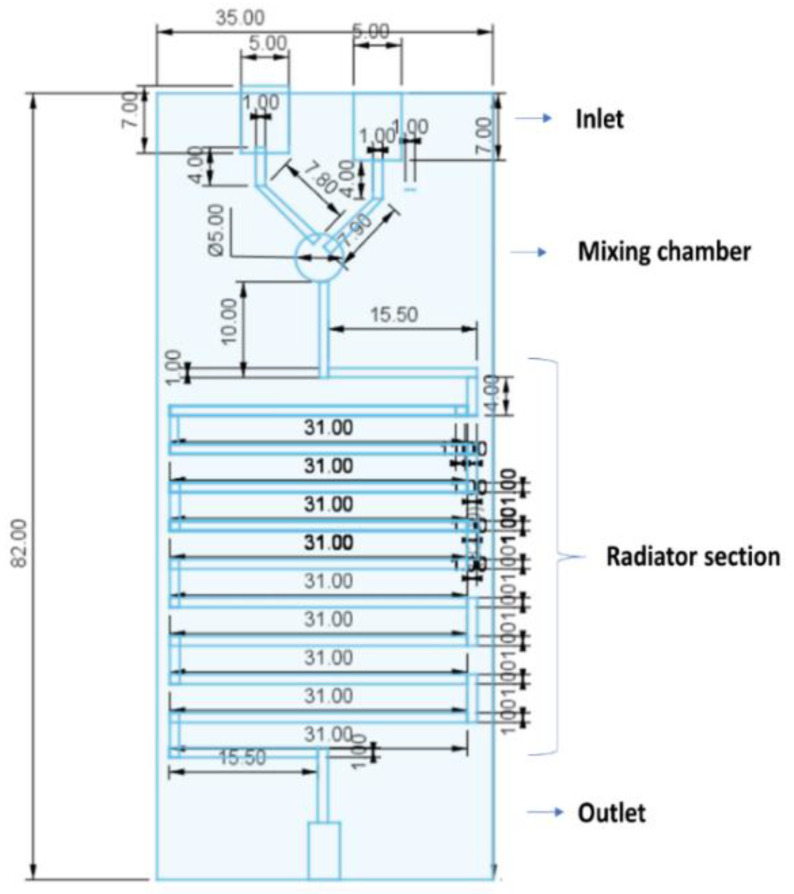
Geometrical design of the 3D printed microfluidic chip (designed using Tinkercad software).

Microfluidic chips were designed with the following dimensions: 82 × 35 × 10 mm, including in one of the extremes, two inputs for the entrance of the solvent and aqueous phase, and in the opposite extreme, one output for the exit of the eluate (Figure 1). Both inputs and output were designed with the same dimensions of 5 mm in diameter and 7 mm in length. The channels through the whole chip were designed with a 1 mm diameter. Liquid flowing from each input channel was forced to join through a 90° angle intersection consisting of a 5 mm diameter circular chamber (mixing chamber). The chamber was followed by a mixing channel designed to mimic a radiator device shape consisting of 11 horizontal columns of 31 mm in length, separated from each other by 4 mm in length connectors. One vertical channels of 15.5 mm in length connected the mixing chamber with the radiator section and a second vertical channel of the same dimensions connected the exit of the radiator with the outlet port. To prevent leakage, a needle tip of 1 mm of diameter was inserted in each input.

**Figure 2:**
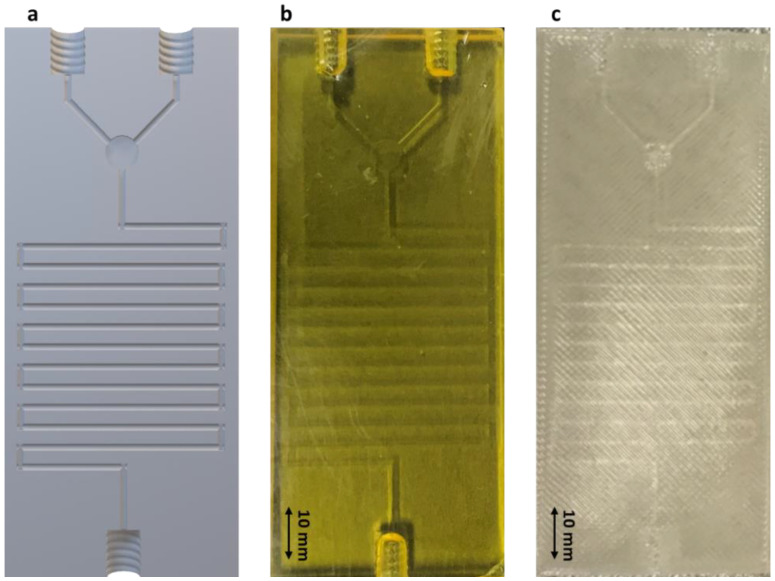
Schematic representation and 3D printed chips. Key: (**a**) Sliced version (.stl file) of the microfluidic chip design; (**b**) Photograph of 3D printed microfluidic chip by SLA; (**c**) Photograph of 3D printed microfluidic chip by FDM. Scale: 10 mm.

**Figure 3:**
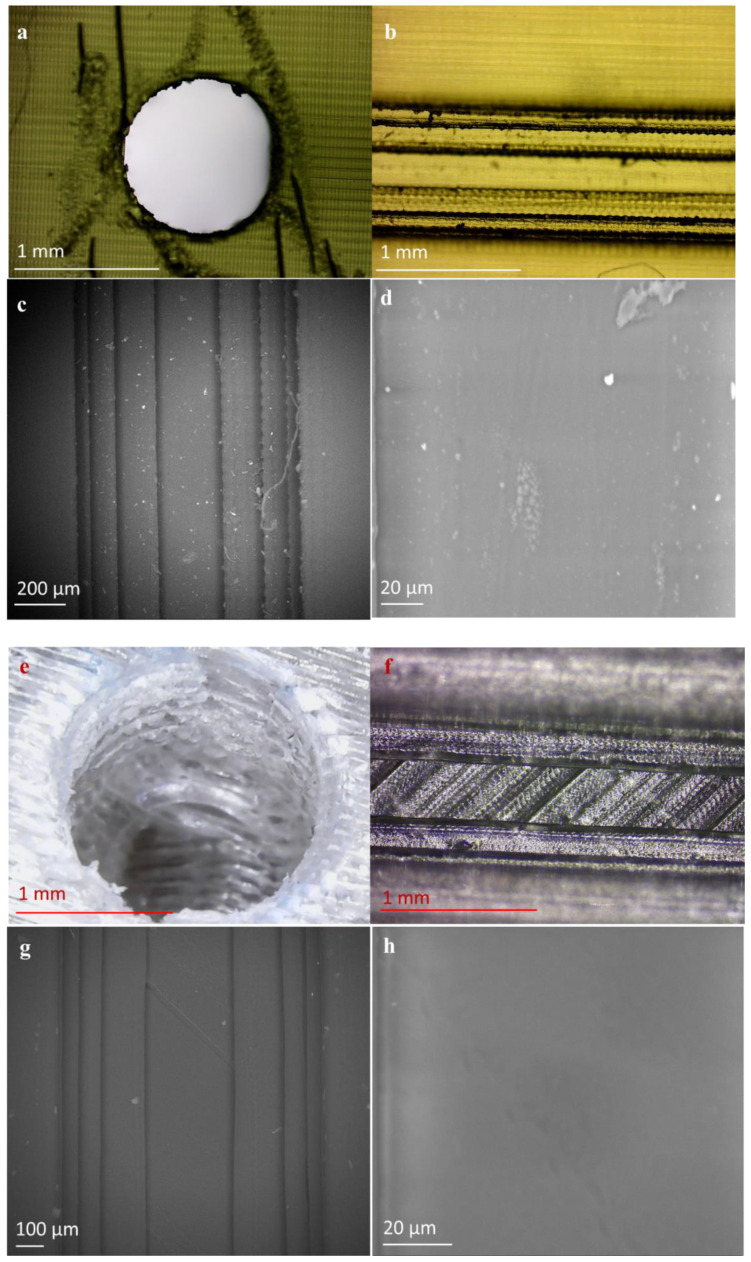
Morphology and channel dimensions visualized with a microscope (4× magnification) and a scanning electron microscope of SLA (**a**–**d**) and FDM (**e**–**h**) printed microfluidic chips.

The geometrical design in Figure 1 was sliced and exported as a .stl file before printing (Figure 2a) and the sliced file was transferred to either the SLA or FDM printer. The polymerization technique was applied to print the chips through SLA, while FDM used a thermoplastic material for printing following the G-code prepared by the slicer. The final 3D printed microfluidic chips are illustrated in Figure 2b,c. The chip geometry matched the .stl file accurately. Both SLA and FDM chips showed no porosity, which is indicative of accurate layer polymerization and deposition, respectively (Figure 3d,h). Chips printed with SLA showed a smoother surface compared to those manufactured by FDM with a channel size of 1 mm in diameter. The dimensions of the channels were measured with a digital microscope ranging from 985–1015 µm (Figure 3). The average surface roughness of the channels was 1.643 ± 0.399 μm and 0.156 ± 0.106 μm for the FDM and SLA printed microfluidics chips, respectively. The Rz value was 7.64 ± 2.15 μm and 0.92 ± 0.53 μm for the FDM and SLA chips, respectively. These results indicate that the channels of the FDM printed chips have a 10.5-fold rougher surfaces than those of the SLA printed chips. The mixing properties of the fluids inside the channels were tested using two different colour liquids (Video link in Supplementary Material).

### 3.2. Microfluidic Preparation and Characterization of NFD Polymeric Nanoparticles

**Figure 4:**
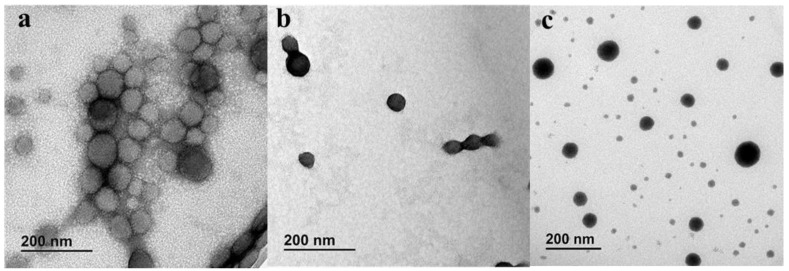
TEM micrographs of NFD polymeric nanoparticles stained with 1% uranyl acetate. Key: (**a**) NFD nanoparticles prepared using FDM printed microfluidic chips; (**b**) NFD nanoparticles prepared using SLA printed microfluidic chips; (**c**) NFD nanoparticles prepared by conventional method. Scale bars: 200 nm.

NFD nanoparticles with quasispherical morphology were obtained from both the SLA and FDM microfluidic chips (Figure 4). Particles obtained from SLA chips showed a smaller mean diameter compared to those prepared from FDM chips. Particle size, polydispersity, and zeta potential were measured for both types of microfluidically produced nanoparticles and the results are summarized in Table 1. NFD nanoparticles manufactured using SLA chips were significantly smaller than those particles obtained from FDM chips, 68 ± 1 nm versus 75 ± 1 nm, respectively (one-way ANOVA, *p* < 0.05), which was closer to the particle size obtained by solvent evaporation. However, the span and PDI were similar in all formulations. No statistically significant differences were observed in the zeta potential values between SLA and FDM nanoparticles or with those fabricated with the conventional method, ranging in all the three cases from −35.5 to −32.5 mV, indicating good colloidal particle stability. In terms of drug encapsulation, NFD nanoparticles showed a 7% greater drug encapsulation when manufactured by SLA than with FDM (one-way ANOA, *p* < 0.05) which was closer to the loading reported by the conventional method.

**Figure 5:**
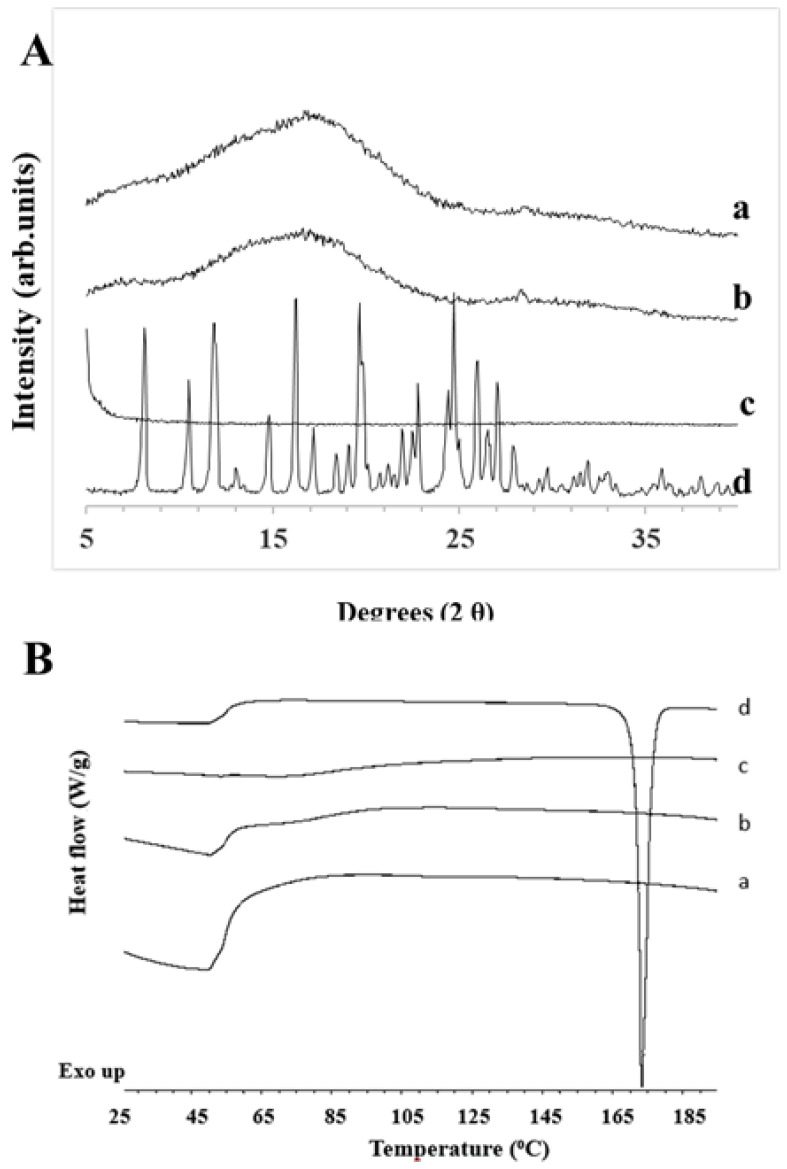
(**A**) XRD patterns and (**B**) DSC thermograms of lyophilized NFD polymeric nanoparticles. Key: (a) NFD polymeric nanoparticles prepared with an SLA printed microfluidic chip; (b) NFD polymeric nanoparticles prepared with FDM printed microfluidic chip; (c) NFD polymeric nanoparticles prepared with conventional evaporation method; (d) Unprocessed NFD.

After lyophilization of microfluidically prepared polymeric nanoparticles, pXRD patterns showed a characteristic amorphous halo for NFD encapsulated within Eudragit nanoparticles manufactured by both SLA and FDM chips, similar to the amorphous halo observed with NFD nanoparticles that were prepared by conventional solvent evaporation. (Figure 5A). In contrast, unprocessed NFD showed characteristic Bragg peaks attributed to the crystalline drug [30]. In the DSC thermograms, the sharp melting event attributed to unprocessed NFD (Figure 5B) disappeared in both nanoparticulate formulations prepared by microfluidic chips and conventional solvent evaporation. These results are aligned with the data obtained from the diffractograms. However, no glass transition of amorphous NFD was observed, which could be due to the overlapping with the dehydration peak that occurred after lyophilization.

**Figure 6:**
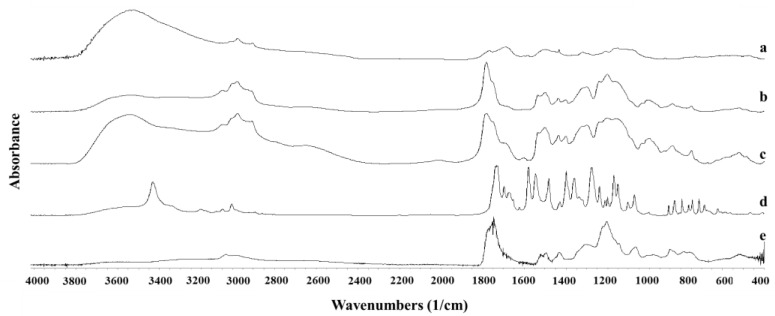
FTIR spectra of NFD polymeric nanoparticles. Key: (a) NFD formulation prepared with conventional evaporation method; (b) NFD formulation prepared with SLA printed microfluidic chip; (c) NFD formulation prepared with FDM printed microfluidic chip; (d) Unprocessed NFD; (e) Eudragit L-100-55.

The FTIR spectra of polymeric nanoparticles and unprocessed NFD are represented in Figure 6. A marked shift in the C=O group of the unprocessed NFD was observed from 1681 cm^−1^ to 1699 cm^−1^ for both NFD polymeric nanoparticles obtained by SLA and FDM chips. A shift was also noticeable in the NO_2_ asymmetric stretch from 1497 to 1484 cm^−1^. Similar bands were obtained with the solvent evaporation method but at a lower intensity. These shifts can explain the amorphous nature of the drug encapsulated within the nanoparticles due to H-bonding interactions with the polymer but also due to the amorphization process during lyophilization. The solid-state molecular interaction between NFD and Eudragit^®^ has been previously characterized by other authors [38]. Shifts in the ester carbonyl group of NFD towards wavelengths close to 1706 cm^−1^ were correlated with a molecular dispersion, while shifts towards the 1701 cm^−1^ wavelength indicated the formation of a phase-separated amorphous suspension between drug and polymer. Based on our FTIR results, NFD will be encapsulated within the core of the Eudragit^®^ nanoparticles, which would explain the shift in the ester carbonyl group of NFD from 1681 to 1699 cm^−1^ and hence, the formation of two separated amorphous domains corresponding to the drug and the polymer.

**Figure 7:**
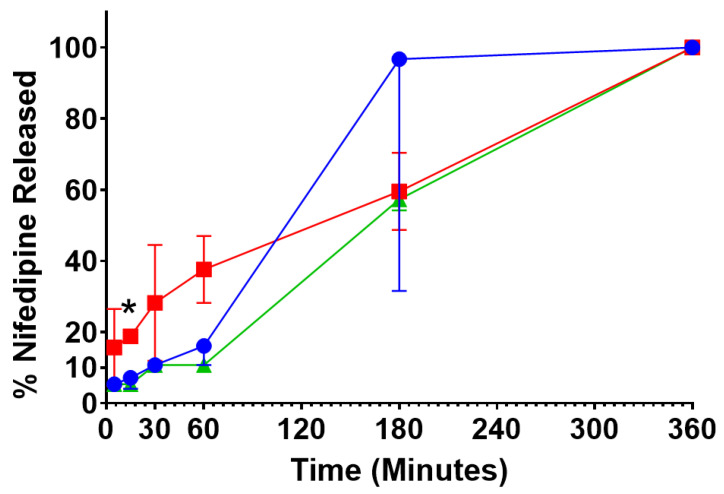
NFD release from polymeric nanoparticulate formulations in simulated gastric fluid (pH 1.2 over 2 h) and simulated intestinal fluid (pH 6.8 over remaining time). Key: (-●-) Polymeric nanoparticles prepared with SLA printed microfluidic chip, (-■-) Polymeric nanoparticles prepared with FDM printed microfluidic chip, (-▲-) Polymeric nanoparticles prepared with the conventional method. A repeated measures ANOVA was undertaken (GraphPad 9) using a Tukey’s post hoc test and indicated that release from FDM microfluidically prepared polymeric nanoparticles was different (* *p* < 0.05) from both SLA microfluidically prepared polymeric nanoparticles as well as those prepared with solvent evaporation.

The release profile was markedly different between NFD nanoparticles manufactured by SLA and FDM chips (*p* < 0.05) (Figure 7). Particles obtained from SLA chips showed a hampered release profile in the acidic media (<20%), while the release increased exponentially when the pH was increased to 6.8. However, particles manufactured by FDM showed a more pronounced burst effect during the first fifteen minutes in acidic media (40%) (*p* < 0.05) followed by a prolonged controlled release during the next five hours. During the first hour, the release profile of NFD nanoparticles prepared by solvent evaporation was similar to that of SLA nanoparticles, followed by a release profile similar to that of NFD nanoparticles prepared with SLA printed microfluidic chips (repeated measures ANOVA, *p* > 0.05). The release of NFD from SLA nanoparticles followed a Hixon–Crowell kinetic model (R^2^ = 0.92), whereas the release from FDM nanoparticles followed a Korsmeyer–Peppas model (R^2^ = 0.98). The different release profiles of NFD can be attributed to different drug-polymer interactions and distribution. The less pronounced burst effect observed from the nanoparticles manufactured by SLA chips can be related to the formation of a thicker polymer shell and higher drug entrapment in the core (explaining the phase-separated amorphous system by FTIR), leading to a greater release at the intestinal pH at which the Eudragit L-100-55 dissolves faster. In contrast, the release profile from FDM nanoparticles could be explained by a more homogenous distribution of the drug within the polymer triggering a burst effect in acidic media (where the drug possesses greater solubility) from the surface initially that reduces over time as the surface is acting as a gel diffusion barrier, which explains why the Korsmeyer Peppas model fits better the kinetics of release. This different distribution can be related to the broader peak at 1699 cm^−1^ observed by FTIR compared to SLA nanoparticles.

## 4. Discussion

The manufacturing of nanomedicines has been demonstrated previously, mostly using commercially available microfluidic chips made of PDMS [39,40]. In this work, we demonstrated that microfluidic chips can be successfully manufactured by 3D printing and can elicit fabricated polymeric nanoparticles similar to other conventional methods such as solvent evaporation [41], with similar morphology and loading and similar release profiles. This pilot study opens the window to the immense possibilities in the manufacturing of nanomedicines. In this work, we have demonstrated that FDM and SLA printers possess the necessary resolution to enable the printing of microfluidic channels of 1 mm in diameter, which are able to be used successfully to fabricate nanomedicines with sizes below 100 nm.

SLA utilizes a photopolymerizable resin that is polymerized layer by layer under the presence of UV light and a photoinitiator to form a solid body. The thickness of the cured layer (CD) is determined by several variables, including the power of the light source, scanning speed, depth of focus, and exposure time, which can be calculated using the following equation:

CD = DP ln (E/EC)(8)

where DP denotes the penetration depth of light, EC denotes the binding energy of the resin, and E denotes the energy power of the light source. Additionally, resin properties are a key factor that affects the quality and performance of 3D printing resolution [26]. To enhance the resolution during SLA printing, a translucent yellow resin was used instead of transparent clear to minimize light bleeding and reduce the penetration depth of light, while maintaining transparency to allow easy visualization of liquid flow within the channels. The binding energy of the resin was optimized by combining a 100% power light source with different exposure times, longer at the bottom layers (60 s) and 3 s for the rest. Longer exposure periods resulted in blocked channels. The overall time for printing SLA chips was 35 min.

On the other hand, FDM printers use thermoplastic filaments as feed material that goes through a hot-melt extruder where the plastic softens enough to be fully inserted in the print head. The molten filament is then deposited layer by layer in the printing area to create the 3D model. The main challenge to overcome when printing microfluidic chips is the poor resolution at the edge and the corner of the channels. The infill of the chip was increased to 100% to reduce leakages and make more robust chips at the expense of necessitating greater amounts of feedstock. In addition, to enhance channel resolution, the layer height was reduced from 0.3 to 0.1 mm and the printing speed was kept to the minimum (10 mm s^−1^ instead of the typical printing speeds, which are 10-fold higher at around 100 mm s^−1^), which increased the printing time per chip to 270 min. The thermoplastic selected material for printing was COC characterized by its resistance to solvents and high transparency, key design factors for microfluidic chips. COC is commonly utilized in food and beverage packaging as well as medical disposables [42]. It has low water absorption (<0.01%) and is electrically insulating. It is also resistant to acids, alcohols, esters, and bases except for non-polar organic solvents (toluene, hexane) [43].

Compared to PDMS chips, 3D printed chips are faster and cheaper to manufacture, especially when using the SLA technique. PDMS requires the fabrication of a mold, while 3D printed microfluidic chips can be engineered directly using worldwide freely available CAD software along with suitable resins and thermoplastic filaments, which are affordable. In this work, channels of 1 mm in diameter were fabricated; however, narrower channels are possible by PDMS, up to 100–200 µm [44]. A diameter of 1 mm was enough to result in adequate mixing, leading to nanoparticles of around 100 nm, which is 3-fold smaller than previous works [45]. Similar particle sizes (100–200 nm) were obtained previously for liposomes when 3D printed microfluidic chips made of polylactic acid using an FDM printer with 600 µm channel diameter were used [46]. Although polymeric nanoparticle manufacture was done in batches in our study, the manufacturing process can be easily converted to a continuous process if the solvent is removed using flow tangential filtration. A statistically significant difference (independent t-test, *p* < 0.05) was observed in the surface roughness of the channels between FDM and SLA chips. SLA led to 10.5-fold smoother internal channels than FDM, which can be correlated with differences in the type of flow experienced inside the channels, resulting in differences in particle size and the release profile of the fabricated nanoparticles [47]. SLA chips led to significantly smaller nanoparticles than those manufactured by FDM chips, and were closer to the size obtained by solvent evaporation considering that smoother channels allow for more uniform and higher fluid velocity.

Microfluidic chips showed a similar outcome to those manufactured by the conventional method in terms of the particles’ release profile. As expected, nanoparticles from the conventional method showed a more gastro-resistant profile in acidic media due to the nature of the selected polymer, which was also similar for particles obtained microfluidically from SLA printed chips. The release from particles prepared with solvent evaporation was sustained in simulated intestinal media. SLA exhibited similar behavior to particles prepared with Eudragit L-100-55 polymers, which was characterized by a hindered release in acidic media followed by a sustained release profile when the pH was raised to 6.8 [48]. FDM nanoparticles showed an initial burst release followed by a sustained release profile over six hours. One possible explanation might be the localization of the drug within the particles and the drug–polymer interaction. The smoother channels of SLA chips lead to a more homogenous loading process where nifedipine is located within the core of the polymeric nanoparticles and results in particles with smaller sizes and a controlled release profile in acid media. However, for particles prepared using FDM printed chips, the drug may be partially located at the surface of the particles, triggering an initial burst release in acid media. Thus, based on our findings, SLA printed microfluidic chips are better in providing manufacturing processes with enhanced mixing properties that are able to elicit particles with optimal drug encapsulation, smaller size, and controlled drug release profile.

## 5. Conclusions

Three-dimensional printed microfluidic chips were successfully designed and manufactured using both SLA and FDM techniques with 1 mm diameter channels and used to fabricate polymeric nanoparticles with good encapsulation efficiencies and particle sizes <100 nm, similar to nanoparticles obtained by solvent evaporation. Prepared nanoparticles exhibited a sustained release profile in the gastrointestinal tract. As nanomedicine development remains costly and time-consuming, 3D printed microfluidic chips can provide a simple method to control the process and convert discontinuous methods into a continuous manufacturing process, which can be easily industrially scalable to produce and maintain key quality attributes of particles.

## Figures and Tables

**Table 1:** Particle characteristics and encapsulation efficiency of NFD polymeric nanoparticles.

Parameters	FDM		SLA		Conventional Method	
	Mean	SD	Mean	SD	Mean	SD
D_10_ (nm)	46	3	41	1	32	1
D_50_ (nm)	75	3	68	2	52	2
D_90_ (nm)	131	12	119	5	91	4
PDI	<0.1	-	<0.1	-	<0.1	-
Span	1.134	0.054	1.153	0.003	1.134	0.002
Zeta Potential (mV)	−35.5	8.1	−32.5	2.7	−35.4	1.8
Encapsulation efficiency (%)	42.3	1.3	49.6	3.2	53.0	2.1

## Data Availability

Data available on request due to restrictions, e.g., privacy or ethical.

## Supplementary Materials

Video of mixing liquids in microfluidic chip https://www.youtube.com/embed/GDm00WhvVyk?feature=oembed (accessed on 6 December 2021).

## References

[1] Kreyling W. (2005). Nanomedicine: An ESF-European Medical Councils (EMRC) forword look report. Strasbg. Cedex.

[2] Research G.V. (2017). Nanomedicine Market Size Worth $350.8 Billion by 2025–GR: 11.2%. https://www.grandviewresearch.com/press-release/global-nanomedicine-market#:~:text=The%20global%20nanomedicine%20market%20is,more%20cost%2Deffective%20than%20traditional.

[3] Shin M.D., Shukla S., Chung Y.H., Beiss V., Chan S.K., Wirth D.M., Chen A., Sack M., Pokorski J.K., Steinmetz N.F. (2020). COVID-19 vaccine development and a potential nanomaterial path forward. Nat. Nanotechnol..

[4] Ventola C.L. (2017). Progress in nanomedicine: Approved and investigational nanodrugs. Pharm. Ther..

[5] Bobo D., Robinson K.J., Islam J., Thurecht K.J., Corrie S.R. (2016). Nanoparticle-Based Medicines: A Review of FDA-Approved Materials and Clinical Trials to Date. Pharm. Res..

[6] Ahmed S., Salmon H., Distasio N., Do H.D., Scherman D., Alhareth K., Tabrizian M., Mignet N. (2021). Viscous Core Liposomes Increase siRNA Encapsulation and Provides Gene Inhibition When Slightly Positively Charged. Pharmaceutics.

[7] Ding S., Anton N., Vandamme T.F., Serra C.A. (2016). Microfluidic nanoprecipitation systems for preparing pure drug or polymeric drug loaded nanoparticles: An overview. Expert Opin. Drug Deliv..

[8] Havel H.A. (2016). Where are the nanodrugs? An industry perspective on development of drug products containing nanomaterials. AAPS J..

[9] Lapworth K., Dawe S., Davis P., Kavanagh D., Young R., Saunders J. (2009). Impulsivity and positive psychotic symptoms influence hostility in methamphetamine users. Addict. Behav..

[10] Committee for Medicinal Products for Human Use (2006). Reflection Paper on Nanotechnology-Based Medicinal Products for Human Use. EMEA/CHMP/79769/2006, London. https://etp-nanomedicine.eu/wp-content/uploads/2018/10/reflection-paper-nanotechnology-based-medicinal-products-human-use_en-1.pdf.

[11] FDA U. (2018). Liposome Drug Products: Chemistry, Manufacturing, and Controls; Human Pharmacokinetics and Bioavailability; and Labeling Documentation. *Guid. Ind.*. https://www.fda.gov/regulatory-information/search-fda-guidance-documents/liposome-drug-products-chemistry-manufacturing-and-controls-human-pharmacokinetics-and.

[12] FDA (2017). Drug Products, Including Biological Products, That Contain Nanomaterials—Guidance for Industry.

[13] Fornaguera C., García-Celma M.J. (2017). Personalized nanomedicine: A revolution at the nanoscale. J. Pers. Med..

[14] Paliwal R., Babu R.J., Palakurthi S. (2014). Nanomedicine scale-up technologies: Feasibilities and challenges. Aaps. Pharm. Sci. Tech..

[15] Sanhai W.R., Sakamoto J.H., Canady R., Ferrari M. (2008). Seven challenges for nanomedicine. Nat. Nanotechnol..

[16] Satalkar P., Elger B.S., Hunziker P., Shaw D. (2016). Challenges of clinical translation in nanomedicine: A qualitative study. Nanomed. Nanotechnol. Biol. Med..

[17] Ventola C.L. (2012). The nanomedicine revolution: Part 1: Emerging concepts. Pharm. Ther..

[18] Au A.V., Beauchamp M.J., Nordin G.P., Woolley A.T. (2016). 3D-Printed Microfluidics. Angew. Chem. Int. Ed..

[19] Wu L.-P., Wang D., Li Z. (2020). Grand challenges in nanomedicine. Mater. Sci. Eng. C.

[20] Ali A., Ahmad U., Akhtar J. (2020). 3D Printing in Pharmaceutical Sector: An Overview. Pharm. Formul. Des.-Recent Pract..

[21] Yuste I., Luciano F.C., González-Burgos E., Lalatsa A., Serrano D.R. (2021). Mimicking bone microenvironment: 2D and 3D in vitro models of human osteoblasts. Pharmacol. Res..

[22] Shah D.M., Morris J., Plaisted T.A., Amirkhizi A.V., Hansen C.J. (2021). Highly filled resins for DLP-based printing of low density, high modulus materials. Addit. Manuf..

[23] Lepowsky E., Tasoglu S. (2018). 3D printing for drug manufacturing: A perspective on the future of pharmaceuticals. Int. J. Bioprinting.

[24] Tiboni M., Campana R., Frangipani E., Casettari L. (2021). 3D printed clotrimazole intravaginal ring for the treatment of recurrent vaginal candidiasis. Int. J. Pharm..

[25] Pereira T., Kennedy J.V., Potgieter J. (2019). A comparison of traditional manufacturing vs. additive manufacturing, the best method for the job. Procedia Manuf..

[26] Hwang H.H., Zhu W., Victorine G., Lawrence N., Chen S. (2018). 3D-printing of functional biomedical microdevices via light-and extrusion-based approaches. Small Methods.

[27] Patwardhan A. (2018). How 3D Printing Will Change the Future of Borrowing Lending and Spending?. Handbook of Blockchain, Digital Finance, and Inclusion.

[28] Choonara Y.E., Toit L.C.d., Kumar P., Kondiah P.P.D., Pillay V. (2016). 3D-printing and the effect on medical costs: A new era?. Expert Rev. Pharm. Outcomes Res..

[29] Ayyoubi S.J.C., Cerda R., Fernández-García P., Knief A., Lalatsa A.M., Healy D.R. (2021). Serrano3D printed spherical mini-tablets: Geometry versus composition effects in controlling dissolution from personalised solid dosage forms. Int. J. Pharm..

[30] Cerda J.R., Arifi T., Ayyoubi S., Kief P., Ballesteros M.P., Keeble W., Barbu E., Healy A.M., Lalatsa A., Serrano D.R. (2020). Personalised 3D printed medicines: Optimising material properties for successful passive diffusion loading of filaments for fused deposition modelling of solid dosage forms. Pharmaceutics.

[31] Smith L.D.R., Serrano M., Mauger F., Bolas-Fernández M.A., Dea-Ayuela A. (2018). Lalatsa Orally bioavailable and effective buparvaquone lipid-based nanomedicines for visceral leishmaniasis. Mol. Pharm..

[32] Serrano D.R., O’Connell P., Paluch K.J., Walsh D., Healy A.M. (2016). Cocrystal habit engineering to improve drug dissolution and alter derived powder properties. J. Pharm. Pharmacol..

[33] Sánchez-Guirales S.A., Jurado N., Kara A., Lalatsa A., Serrano D.R. (2021). Understanding direct powder extrusion for fabrication of 3D printed personalised medicines: A case study for nifedipine minitablets. Pharmaceutics.

[34] Costa P., Sousa Lobo J.M. (2001). Modeling and comparison of dissolution profiles. Eur. J. Pharm. Sci..

[35] Siepmann J., Siepmann F. (2013). Mathematical modeling of drug dissolution. Int. J. Pharm..

[36] Lao L.L., Peppas N.A., Chiang BOey F.Y., Venkatraman S.S. (2011). Modeling of drug release from bulk-degrading polymers. Int. J. Pharm..

[37] Mamani P.L., Ruiz-Caro R., Veiga M.D. (2012). Matrix tablets: The effect of hydroxypropyl methylcellulose/anhydrous dibasic calcium phosphate ratio on the release rate of a water-soluble drug through the gastrointestinal tract I. In Vitro tests. Aaps. Pharm. Sci. Tech..

[38] Huang J., Li Y., Wigent R.J., Malick W.A., Sandhu H.K., Singhal D., Shah N.H. (2011). Interplay of formulation and process methodology on the extent of nifedipine molecular dispersion in polymers. Int. J. Pharm..

[39] Kastner E., Verma V., Lowry D., Perrie Y. (2015). Microfluidic-controlled manufacture of liposomes for the solubilisation of a poorly water soluble drug. Int. J. Pharm..

[40] Roces C.B., Khadke S., Christensesn D., Perrie Y. (2019). Scale-Independent Microfluidic Production of Cationic Liposomal Adjuvants and Development of Enhanced Lymphatic Targeting Strategies. Mol. Pharm..

[41] SadAbadi H., Badilescu S., Packirisamy M., Wuthrich R. (2013). Integration of gold nanoparticles in PDMS microfluidics for lab-on-a-chip plasmonic biosensing of growth hormones. Biosens. Bioelectron..

[42] Bruijns B., Veciana A., Tiggelaar R., Gardeniers H. (2019). Cyclic Olefin Copolymer Microfluidic Devices for Forensic Applications. Biosensors.

[43] Nunes P.S., Ohlsson P.D., Ordeig O., Kutter J.P. (2010). Cyclic olefin polymers: Emerging materials for lab-on-a-chip applications. Microfluid. Nanofluidics.

[44] Chen Z., Han J.Y., Shumate L., Fedak R., Devoe D.L. (2019). High Throughput Nanoliposome Formation Using 3D Printed Microfluidic Flow Focusing Chips. Adv. Mater. Technol..

[45] Ballacchino G., Weaver E., Mathew E., Dorati R., Genta I., Conti B., Lamprou D.A. (2021). Manufacturing of 3D-Printed Microfluidic Devices for the Synthesis of Drug-Loaded Liposomal Formulations. Int. J. Mol. Sci..

[46] Tiboni M., Benedetti S., Skouras A., Curzi G., Perinelli D.R., Palmieri G.F., Casettari L. (2020). 3D-printed microfluidic chip for the preparation of glycyrrhetinic acid-loaded ethanolic liposomes. Int. J. Pharm..

[47] Lee J.M., Zhang M., Yeong W.Y. (2016). Characterization and evaluation of 3D printed microfluidic chip for cell processing. Microfluid. Nanofluidics.

[48] Jog R., Unachukwu K., Burgess D.J. (2016). Formulation design space for stable, pH sensitive crystalline nifedipine nanoparticles. Int. J. Pharm..

